# *Notes from the Field:* Zoonotic *Mycobacterium bovis* Disease in Deer Hunters — Michigan, 2002–2017

**DOI:** 10.15585/mmwr.mm6837a3

**Published:** 2019-09-20

**Authors:** James Sunstrum, Adenike Shoyinka, Laura E. Power, Daniel Maxwell, Mary Grace Stobierski, Kim Signs, Jennifer L. Sidge, Daniel J. O’Brien, Suelee Robbe-Austerman, Peter Davidson

**Affiliations:** ^1^Beaumont Health System, Dearborn, Michigan; ^2^Department of Epidemiology, School of Public Health, University of Michigan, Ann Arbor; ^3^Department of Internal Medicine, Division of Infectious Diseases, University of Michigan Medical School, Ann Arbor; ^4^MidMichigan Health, Alpena, Michigan; ^5^Michigan Department of Health and Human Services; ^6^Wildlife Disease Laboratory, Michigan Department of Natural Resources, Lansing; ^7^National Veterinary Services Laboratories, Animal and Plant Health Inspection Service, U.S. Department of Agriculture, Ames, Iowa.

In May 2017, the Michigan Department of Health and Human Services was notified of a case of pulmonary tuberculosis caused by *Mycobacterium bovis* in a man aged 77 years. The patient had rheumatoid arthritis and was taking 5 mg prednisone daily; he had no history of travel to countries with endemic tuberculosis, no known exposure to persons with tuberculosis, and no history of consumption of unpasteurized milk. He resided in the northeastern Lower Peninsula of Michigan, which has a low incidence of human tuberculosis but does have an enzootic focus of *M. bovis* in free-ranging deer (*Odocoileus virginianus*). The area includes a four-county region where the majority of *M. bovis*–positive deer in Michigan have been found (*1*). Statewide surveillance for *M. bovis* via hunter-harvested deer head submission has been ongoing since 1995 (*1*); in 2017, 1.4% of deer tested from this four-county region were culture-positive for *M. bovis*, compared with 0.05% of deer tested elsewhere in Michigan (*2*). The patient had regularly hunted and field-dressed deer in the area during the past 20 years. Two earlier hunting-related human infections with *M. bovis* were reported in Michigan in 2002 and 2004. In each case, the patients had signs and symptoms of active disease and required medical treatment.

Whole-genome sequencing of the patient’s respiratory isolate was performed at the National Veterinary Services Laboratories in Ames, Iowa. The isolate was compared against an extensive *M. bovis* library, including approximately 900 wildlife and cattle isolates obtained since 1993 and human isolates from the state health department. This 2017 isolate had accumulated one single nucleotide polymorphism compared with a 2007 deer isolate (Figure), suggesting that the patient was exposed to a circulating strain of *M. bovis* at some point through his hunting activities and had reactivation of infection as pulmonary disease in 2017.

**FIGURE:**
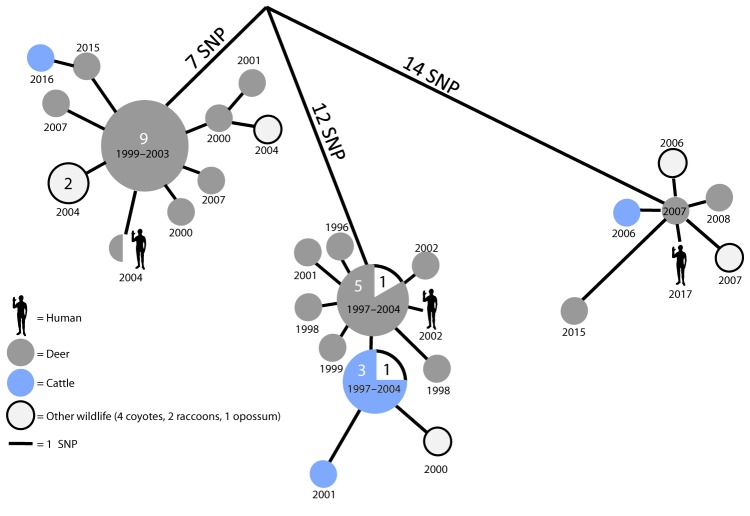
Phylogenetic analysis of the most closely related *Mycobacterium bovis* isolates associated with human tuberculosis cases* — Michigan, 2002–2017 **Abbreviation**: SNP = single nucleotide polymorphism. * The most recent common ancestor is thought to have spilled over into the local deer population from livestock at least one time during 1940–1960. All human cases are within 0–1 SNP of sharing a common ancestor with a deer isolate. Numerals denote multiple identical isolates (for example, identical *M. bovis* isolates from 5 deer and 1 other animal).

Whole-genome sequencing also was performed on archived specimens from two hunting-related human *M. bovis* infections diagnosed in 2002 (pulmonary) and 2004 (cutaneous) that were epidemiologically and genotypically linked to deer (*3*). The 2002 human isolate had accumulated one single nucleotide polymorphism since sharing an ancestral genotype isolated from several deer in Alpena County, Michigan, as early as 1997; the 2004 human isolate shared an identical genotype with a grossly lesioned deer harvested by the patient in Alcona County, Michigan, confirming that his infection resulted from a finger injury sustained during field-dressing. The 2002 and 2017 cases of pulmonary disease might have occurred following those patients’ inhalation of aerosols during removal of diseased viscera while field-dressing deer carcasses (*4*).

In Michigan, deer serve as maintenance and reservoir hosts for *M. bovis*, and transmission to other species has been documented (*1*). Since 1998, 73 infected cattle herds have been identified in Michigan (*5*), resulting in increased testing and restricted movement of cattle outside the four-county zone. Transmission to humans also occurs, as demonstrated by the three cases described in this report; however, the risk for transmission is understudied. Similar to *Mycobacterium tuberculosis*, exposure to *M. bovis* can lead to latent or active infection, with risk for eventual reactivation of latent disease, especially in immunocompromised hosts. To prevent exposure to *M. bovis* and other diseases, hunters are encouraged to use personal protective equipment while field-dressing deer. In addition, hunters in Michigan who submit deer heads* that test positive for *M. bovis* might be at higher risk for infection, and targeted screening for tuberculosis could be performed.^†^ Close collaboration between human and animal health sectors is essential for containing this zoonotic infection.

## Footnotes

* https://www.michigan.gov/dnr/0,4570,7-350-79119_79147_81438---,00.html.

† https://www.cdc.gov/tb/publications/factsheets/general/mbovis.htm.
